# Poor Bone Quality is Associated With Greater Arterial Stiffness: Insights From the UK Biobank

**DOI:** 10.1002/jbmr.4164

**Published:** 2020-09-22

**Authors:** Zahra Raisi-Estabragh, Luca Biasiolli, Jackie Cooper, Nay Aung, Kenneth Fung, José M Paiva, Mihir M Sanghvi, Ross J Thomson, Elizabeth Curtis, Julien Paccou, Jennifer J Rayner, Konrad Werys, Henrike Puchta, Katharine E Thomas, Aaron M Lee, Stefan K Piechnik, Stefan Neubauer, Patricia B Munroe, Cyrus Cooper, Steffen E Petersen, Nicholas C Harvey

**Affiliations:** 1 William Harvey Research Institute, National Institute for Health Research (NIHR) Barts Biomedical Research Centre, Queen Mary University of London, Charterhouse Square, London, UK; 2 Barts Heart Centre, St Bartholomew’s Hospital, Barts Health National Health Service (NHS) Trust, London, UK; 3 Division of Cardiovascular Medicine, Radcliffe Department of Medicine, National Institute for Health Research Oxford Biomedical Research Centre, University of Oxford, Oxford, UK; 4 Medical Research Council (MRC) Lifecourse Epidemiology Unit, University of Southampton, Southampton, UK; 5 Rheumatology Department, Lille University Hospital, CHU Lille, MABlab ULR 4490, 59037 Lille, France; 6 National Institute for Health Research (NIHR) Southampton Biomedical Research Centre, University of Southampton and University Hospital Southampton National Health Service (NHS) Foundation Trust, Southampton, UK; 7 National Institute for Health Research (NIHR) Oxford Biomedical Research Centre, University of Oxford, Oxford, UK; 8 Department of Cardiology, Royal Free Hospital, Royal Free London NHS Foundation Trust, London, UK

**Keywords:** Arterial Stiffness, Cardiovascular Disease, Epidemiology, Ischemic Heart Disease, Osteoporosis

## Abstract

Osteoporosis and ischemic heart disease (IHD) represent important public health problems. Existing research suggests an association between the two conditions beyond that attributable to shared risk factors, with a potentially causal relationship. In this study, we tested the association of bone speed of sound (SOS) from quantitative heel ultrasound with (i) measures of arterial compliance from cardiovascular magnetic resonance (aortic distensibility [AD]); (ii) finger photoplethysmography (arterial stiffness index [ASI]); and (iii) incident myocardial infarction and IHD mortality in the UK Biobank cohort. We considered the potential mediating effect of a range of blood biomarkers and cardiometabolic morbidities and evaluated differential relationships by sex, menopause status, smoking, diabetes, and obesity. Furthermore, we considered whether associations with arterial compliance explained association of SOS with ischemic cardiovascular outcomes. Higher SOS was associated with lower arterial compliance by both ASI and AD for both men and women. The relationship was most consistent with ASI, likely relating to larger sample size available for this variable (*n* = 159,542 versus n = 18,229). There was no clear evidence of differential relationship by menopause, smoking, diabetes, or body mass index (BMI). Blood biomarkers appeared important in mediating the association for both men and women, but with different directions of effect and did not fully explain the observed effects. In fully adjusted models, higher SOS was associated with significantly lower IHD mortality in men, but less robustly in women. The association of SOS with ASI did not explain this observation. In conclusion, our findings support a positive association between bone and vascular health with consistent patterns of association in men and women. The underlying mechanisms are complex and appear to vary by sex.

## Introduction

Osteoporosis is a significant public health problem, particularly in aging populations. In the UK, approximately one in three women and one in five men will sustain an osteoporotic fracture in their lifetime.^(1)^ Ischemic heart disease (IHD) is the most common cause of morbidity and mortality in the world.^(2)^

Osteoporosis and atherosclerosis share a number of risk factors, such as older age, smoking, and sedentary lifestyle. Interestingly, several studies have shown an association between the two conditions beyond these shared risk factors.^(3–6)^ Additionally, biological and genetic studies have proposed common mechanisms driving bone mineralization and atherogenesis.^(7–9)^ Overall, there is evidence for common causal pathways linking the two disease processes. However, existing literature is limited by small sample sizes, lack of objective measures of bone and heart health, and inability to adequately consider potential mediators and confounders. Further, although sex differential disease patterns and the modifying effect of menopause on bone and cardiovascular health are well-recognized, such distinctions have not been clearly elucidated with regard to relationships between these two disease areas.

We studied, in the UK Biobank (UKB), the association of speed of sound (SOS) assessed by quantitative heel ultrasound with measures of arterial compliance on cardiovascular magnetic resonance (CMR) imaging and finger photoplethysmography. We considered the potential mediating effect of a range of blood biomarkers and cardiometabolic morbidities and evaluated differential relationships by sex, menopause status, smoking, diabetes, and obesity. Furthermore, we considered the importance of this relationship in explaining association of SOS with ischemic cardiovascular outcomes.

## Subjects and Methods

### Setting and recruitment

The UKB is a population study incorporating over half a million participants recruited between 2006 and 2010 from across the UK.^(10)^ Individuals aged 40 to 69 years were identified through National Health Service (NHS) registers and invited to participate. The baseline assessment included detailed review of demographics, lifestyle, medical history, a series of physical measures, and blood sampling. The protocol is publicly available.^(11)^ Individuals who were unable to consent or complete baseline assessment due to illness or discomfort were not recruited. Linkages with Hospital Episode Statistics (HES) and death registers enable longitudinal tracking of health outcomes for all participants. Additionally, UKB has produced algorithmically defined outcomes for incidence of key illnesses through checks across multiple data sources.^(12)^ The UKB Imaging Study, which includes cardiovascular magnetic resonance (CMR) imaging, aims to image a subset of 100,000 participants; since its launch in 2015, over 48,000 (July 2020) participants have been scanned.^(13)^

### Ethics

This study was covered by the ethical approval for UK Biobank studies from the NHS National Research Ethics Service on June 17, 2011 (Ref 11/NW/0382) and extended May 10, 2016 (Ref 16/NW/0274).

### Calcaneal quantitative ultrasound

Calcaneal quantitative ultrasound (QUS) is a noninvasive and radiation-free method of assessing bone quality. QUS parameters are good predictors of fragility fractures and correlate reliably with bone mineral density (BMD) measured by dualenergy x-ray absorptiometry (DXA).^(14,15)^

Calcaneal QUS was performed for the whole UKB cohort at baseline using the Sahara Clinical Bone Sonometer (Hologic, Inc., Marlborough, MA, USA) according to a predefined standard operating procedure (SOP).^(16)^ Daily quality control checks of the sonometer were performed using a phantom. Measurement was not taken for individuals with open wounds around the heel or metal implants in the heel.

**Fig 1:**
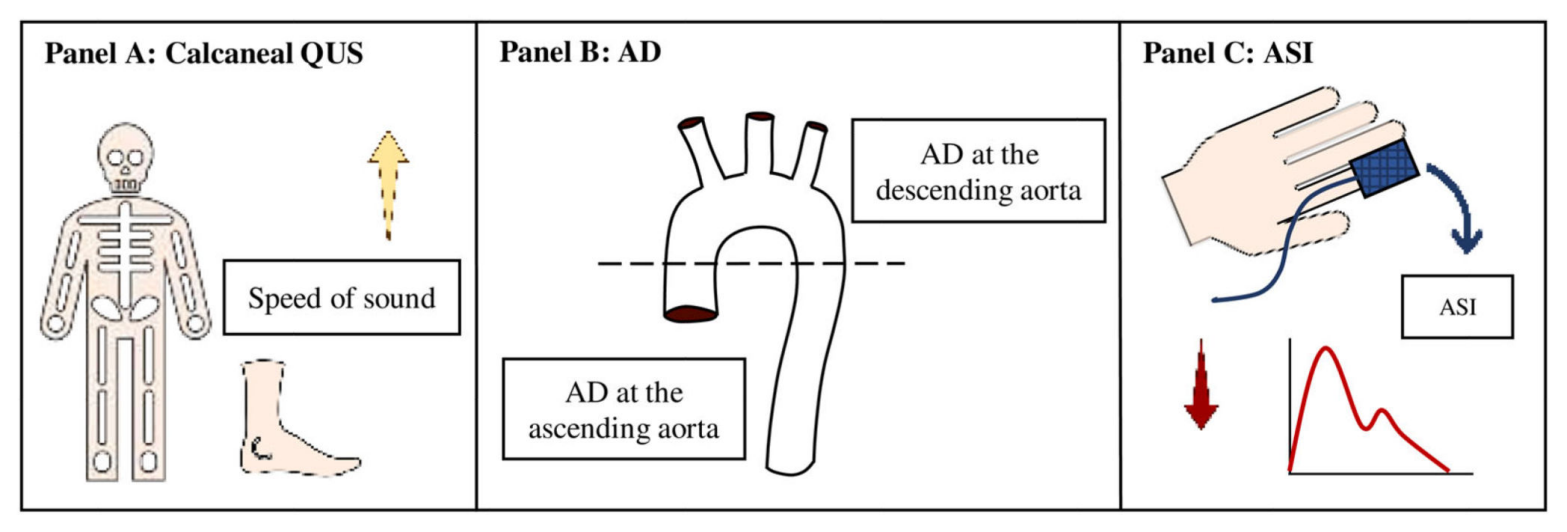
Overview of the measures of bone and vascular health used in the study. (*A*) Higher speed of sound measured on calcaneal quantitative ultrasound indicates better bone health. (*B*) Aortic distensibility provides direct estimates of local aortic stiffness. Higher aortic distensibility at the ascending/descending aorta measured by cardiovascular magnetic resonance indicates better vascular health. (*C*) Arterial stiffness index estimates stiffness in the large arteries. Lower arterial stiffness index by finger photoplethysmography indicates lower stiffness in the large arteries and better vascular health. Arrows indicate direction of parameter associated with better bone/vascular health. AD = aortic distensibility; ASI = arterial stiffness index; QUS = quantitative ultrasound.

The device automatically generates two parameters: SOS and broadband ultrasound attenuation (BUA). SOS measures the speed at which ultrasound travels through bone; it is calculated by dividing the ultrasound transit time by the length of body part studied. BUA is the slope between the attenuation of sound signal and its frequency as it travels through the bone and soft tissue. Higher SOS and BUA values indicate better bone health (Fig. 1A). Within UKB, if BUA data were missing, it was estimated from the SOS measure. We therefore used SOS in this analysis because it was always directly measured. In cases where bilateral measurements were available, we used the mean.

### Arterial stiffness

Arterial stiffness is a measure of vascular compliance; increased stiffness indicates adverse remodeling of the medial layer and impairment of arterial bioelastic function. Greater arterial stiffness indicates higher risk of atherosclerotic disease and has been validated in a variety of settings.^(17,18)^

#### Arterial stiffness index

The arterial stiffness index (ASI) is an indirect estimate of large artery stiffness derived from the contour of a pulse waveform as it propagates and is reflected within the arterial tree.^(17)^ Higher ASI represents greater stiffness in the large arteries and is associated with adverse ischemic cardiovascular outcomes.^(18)^ Lower ASI indicates greater arterial compliance and better vascular health (Fig. 1C).

**Fig 2:**
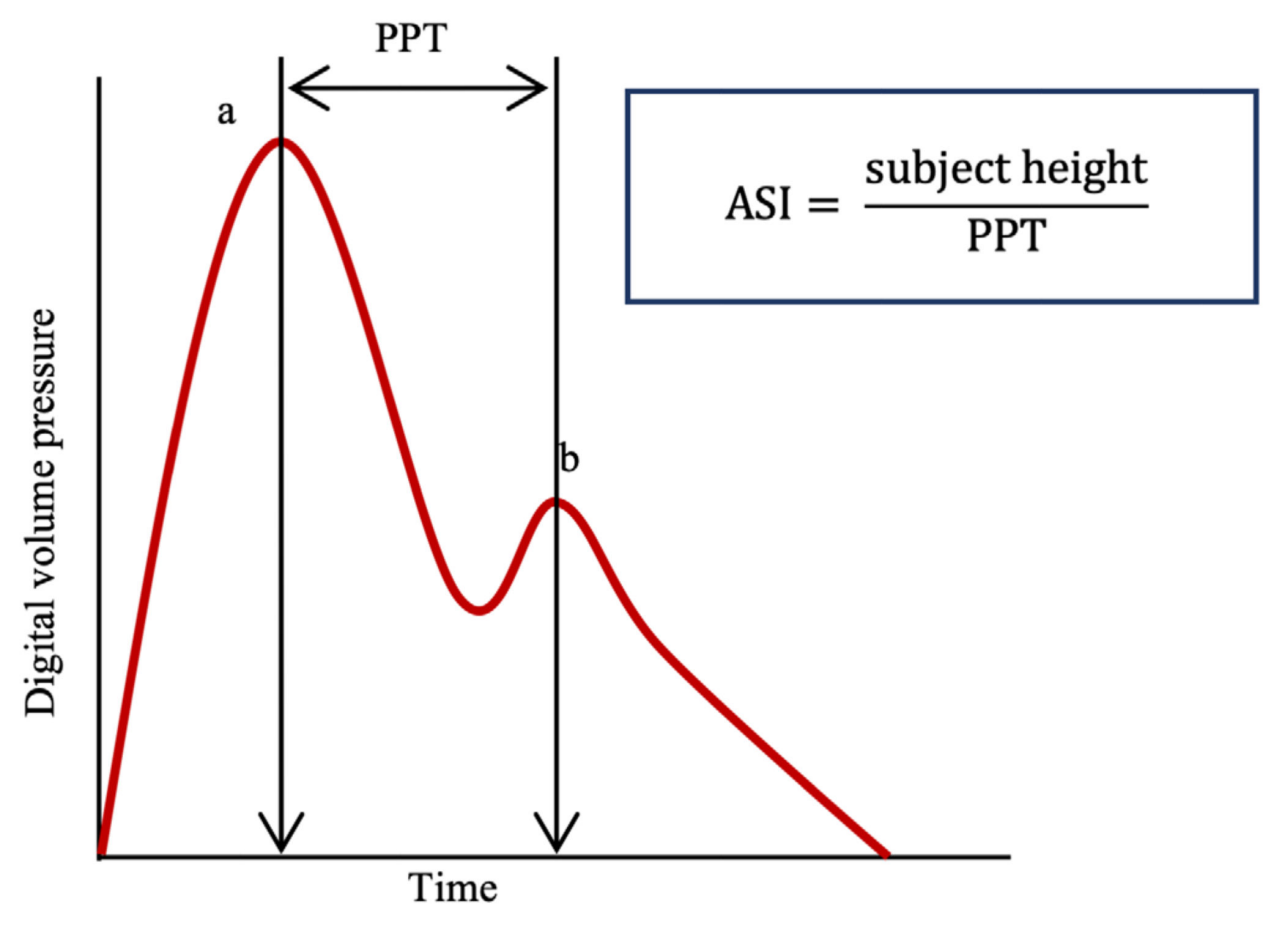
Typical digital volume pressure waveform from PulseTrace sensor. Arterial stiffness index (ASI) is calculated by dividing the subject height by the time between the systolic (a) and diastolic (b) peaks. PPT = peak-to-peak time.

ASI was measured at the baseline visit using finger photoplethysmography with the PulseTrace PCA2 (CareFusion, San Diego, CA, USA) device in accordance with a predefined SOP.^(19)^ The participant was seated, and restrictive clothing removed from the upper arm. The PulseTrace infrared sensor was clipped onto a finger and measurement taken over 10 to 15 s. The device provides a pulse waveform, which demonstrates a systolic and diastolic peak, and the time delay between the two peaks (Fig. 2). The peak-to-peak time (PPT) represents the transit time for the pulse wave from the root of the subclavian artery to the point of reflection and back. The stiffer the large arteries, the quicker the transit time (shorter PPT). The path length for the pulse is proportional to the height of the individual; therefore, an ASI may be calculated by dividing height by PPT (Fig. 2). ASI (m/s) was measured for 169,791 participants at baseline.

#### Aortic distensibility

Aortic distensibility (AD) is a direct measure of local arterial stiffness determined by the change in aortic cross-sectional area in systole-diastole (ie, aortic strain) divided by central pulse pressure (CPP in mmHg).^(20)^ AD is calculated using the formula in Equation (1). Higher AD indicates a more compliant aorta and better vascular health (Fig. 1B).

(1) $$AD=\frac{A_{\mathrm{MAX}}-A_{\mathrm{MIN}}}{A_{\mathrm{MIN}}\times CPP}$$

where A_max_ is the maximal and A_min_ is the minimal aortic lumen area (mm^2^).

**Fig 3:**
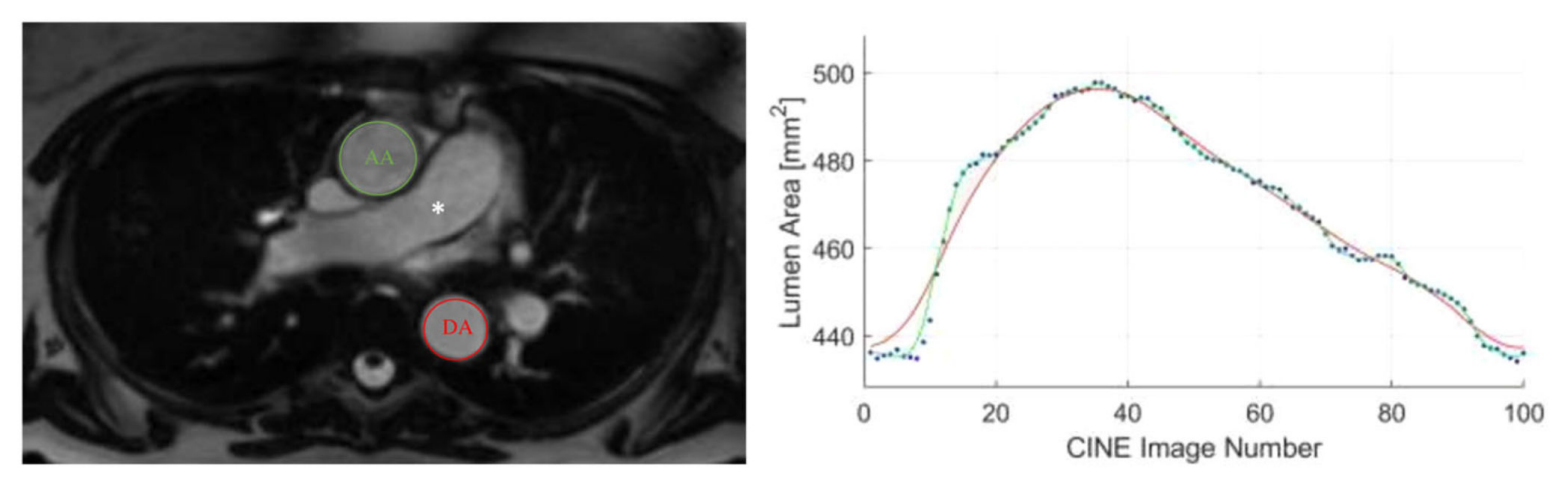
Measurement of aortic distensibility from cardiac magnetic resonance images. Aortic distensibility determined by considering systolic-diastolic variation in cross-sectional lumen area of the AA and DA measured at the level of the main pulmonary artery (*) on transverse cine cardiac magnetic resonance images. The transverse magnetic resonance image (left) was acquired as part of the UK Biobank imaging study; annotations have been added and image used by kind permission of UK Biobank©. AA = ascending aorta; DA = descending aorta.

AD was measured on cine CMR images showing transverse cross-sections of the ascending and descending aorta throughout the cardiac cycle (Fig. 3). A fully automated image analysis workflow has been developed and validated on a large subset of UKB studies (n = 5065).^(21)^ The analysis pipeline has been propagated to cover the first 20,000 UKB CMR scans.

### Cardiovascular outcomes

We considered outcomes occurring from point of recruitment (2006–2010) to the latest UKB censor dates (mortality outcomes: January 31, 2018; incident AMI: March 31, 2017) giving follow-up duration of 7 to 12 years. IHD mortality was defined as primary cause of death attributed to IHD on death registration documents. Incident acute myocardial infarction (AMI) was derived from algorithmically defined outcomes, which includes HES and death register data^(22)^; AMIs occurring after the baseline visit were considered.

### Definition of covariates

Age, sex, and ethnicity were taken as recorded at baseline. Smoking and alcohol intake were defined according to self-report at baseline. Material deprivation is recorded in the UKB as the Townsend index. We calculated a continuous measure for level of physical activity in metabolic equivalent (MET) minutes/week by weighting different types of activity (walking, moderate, or vigorous) by its energy requirements as per the International Physical Activity Questionnaire (IPAQ) study.^(23)^ Hypertension, diabetes, hypercholesterolemia, and menopause were defined based on self-report at baseline. Body mass index (BMI) was calculated from height and weight recorded at baseline. The following serum biochemistry measures (from bloods collected at the baseline visit) were considered as potential mediators: C reactive protein (CRP); creatinine; vitamin D; calcium; alkaline phosphatase (ALP); insulin-like growth factor 1 (IGF1); sex hormone binding globulin (SHBG); testosterone; testosterone/SHBG; estradiol; phosphate, and cystatin C.

### Statistical analysis

Statistical analysis was performed using R studio version 3.6.0 (R Studio, Boston, MA, USA; https://rstudio.com/ [open source]; R Foundation for Statistical Computing, Vienna, Austria; https://www.r-project.org/) and Stata version 14 (StataCorp LP, College Station, TX, USA). Continuous variables are summarized as mean (standard deviation [SD]) for normally distributed parameters and median (interquartile range [IQR]) for skewed distributions. We used a 1.5× IQR rule to remove outliers from the SOS, ASI, and AD variables.

We tested the association of SOS with the ischemic cardiovascular outcomes separately for men and women using competing risk regression models.^(24)^ We report subdistribution hazard ratios (SHRs) per 1 SD increase in SOS with the corresponding 95% confidence intervals (CIs) and *p* values. We next considered the association of SOS with measures of arterial compliance (ASI, AD) using multivariate linear regression models adjusting for age, exercise, smoking, material deprivation, alcohol intake, hypercholesterolemia, diabetes, and hypertension. Results are presented as SD change in vascular measure per 1 SD increase in SOS, tested separately by sex. We checked for nonlinearity of this relationship using restricted cubic splines. In addition, we performed subgroup analyses by menopause (women only), smoking status, diabetes, and obesity. We tested whether associations of SOS with vascular compliance explained relationships with ischemic cardiovascular outcomes.

Finally, we evaluate the mediating effect of a range of blood biomarkers (CRP, creatinine, vitamin D, calcium, ALP, IGF1, SHBG, testosterone, testosterone/SHBG, estradiol, phosphate, cystatin C) and cardiometabolic morbidities (hypertension, diabetes, hypercholesterolemia) selected based on evidence outlined in existing cardiovascular disease literature. The mediating effect of each mediator was first tested individually, if a significant effect was detected (p < .003, corrected for 15 mediators), the mediator was taken forward for multiple mediation analysis. Independent indirect effects were calculated for each mediator as described by Van Der Weele and Vansteelandt.^(25)^ CIs were constructed using bootstrap resampling. We thus calculated the direct and indirect effect of each mediator and present the proportion of effect mediated as a percentage of the total effect.

## Results

### Baseline characteristics

Complete data for SOS and ASI were available for 71,949 men and 87,593 women (Table 1). Average age was 58 years (range, 50–63 years). Rates of smoking, hypertension, diabetes, and high cholesterol were 28.0%, 5.6%, and 19.7%, respectively. Men had a poorer cardiometabolic profile compared to women. The majority of women (73.0%) were postmenopause. There were 18,229 participants with SOS and AD data; their baseline characteristics are summarized in Supplementary Table 1.

### Association of SOS with measures of arterial stiffness

In fully adjusted linear regression models, higher SOS was associated with lower ASI; the relationship appeared significant and of similar magnitude for both men and women (Table 2). Higher SOS was associated with greater AD at the ascending aorta in fully adjusted models for women, but not for men. Higher SOS was associated with greater AD at the descending aorta in fully adjusted models for men, but not for women. There was no evidence of nonlinearity for these relationships (Supplementary Table 2). There were no significant differences in the relationships between men and women.

In stratified analyses, we did not find a differential pattern of association by menopause (Table 3). Higher SOS was associated with lower ASI in premenopausal and postmenopausal women. There was loss of statistical significance in the AD associations, likely due to smaller sample size, again with no evidence of differential relationship by menopause.

### Association of SOS with measures of arterial stiffness by smoking status, diabetes, and BMI

We found no significant difference in pattern of associations in subgroup analysis by smoking status (Supplementary Table 3). Subgroup analysis by diabetes appeared to show a differential relationship with greater effect in nondiabetics; the interaction term was significant for the relationship between SOS and ASI in men (*p* = .012) (Supplementary Table 4). With regard to BMI (Supplementary Table 5), there appeared to be differential effect of SOS on ASI in men with BMI in the normal or overweight categories versus those in the obese category with a significant interaction term (p = .0008). These findings should be interpreted with caution, given the different sizes and composition of the subcohorts.

### Mediation analysis

We considered the role of mediators in the relationship between SOS and ASI because this appeared the most consistent relationship in previous analyses. We considered, separately for men and women, potential mediating effects of the following variables: CRP, creatinine, vitamin D, calcium, ALP, IGF1, SHBG, testosterone, testosterone/SHBG, estradiol, phosphate, cystatin C, hypertension, diabetes, and hypercholesterolemia. We first checked the mediating effect of each variable individually (Supplementary Tables 6 and 7); variables with significant mediated effects were taken forward for multiple mediation analysis. In the final models, we included variables that had statistically significant effects in the multiple mediator model (Supplementary Tables 8 and 9).

In multiple mediation analysis, biomarkers relating to bone mineralization appeared important for both men and women. For men, ALP, phosphate, and vitamin D accounted for 7.5%, 4.6%, and 3.2%, respectively, of the observed effect. In women, ALP and phosphate accounted for 9.6% and 13.2%, respectively, of the observed effect. CRP accounted for 6.1% of the mediated effect in men and −8.6% in women. SHBG had an important suppressing effect for both men and women; adjustment for this variable increased the effect by 17.14% and 19.55%, respectively.

In men the overall effect was mediation; ie, the magnitude of the main exposure-outcome relationship effect was reduced by adjustment for the mediators. In the women the effect was one of suppression rather than mediation as the magnitude of the exposure-outcome relationship increased when we added the potential mediators. The association between ASI and SOS remained significant with all the mediators in the model.

### Association of SOS with ischemic cardiovascular outcomes

SOS was available for 477,683 participants at baseline. We considered association with IHD mortality and incident AMI for this cohort (Table 4); baseline characteristics are summarized in Supplementary Table 10. Follow-up time for mortality was 2,342,445 person years for women and 1,888,767 for men. There were 388 IHD deaths in women (rate = 0.17 per 1000 person years) and 1722 (rate = 0.91 per 1000 person years) in men. Followup time for incident AMI was 2,123,170 person years for women and 1,659,850 person years for men. During this time, there were 2415 AMI events in women (rate = 1.14 per 1000 person years) and 5616 events (rate = 3.38 per 1000 person years) in men.

In crude models including age only, higher SOS was associated with significantly reduced hazard of both incident AMI and IHD mortality in men, but with weaker associations in women. There was attenuation in the relationship with incident AMI with addition of exercise, material deprivation, and alcohol to the model. The negative association with IHD mortality remained in men in this model and in a further model additionally including hypertension, hypercholesterolemia, and diabetes. In this fully adjusted model, for men, 1 SD increase of SOS was associated with 14% lower hazard of IHD mortality (SHR 0.86; 95% CI, 0.75 to 1.00; *p* = 4.0 × 10^−7^).

We tested whether the relationship of SOS with IHD mortality may be explained by observed associations of the former with ASI. Addition of ASI as covariate to competing risk models did not alter the association of greater SOS with lower IHD mortality in men, or women (Supplementary Table 11). Therefore, it appeared that the association with IHD mortality is likely to occur through other mechanisms.

## Discussion

### Summary of study findings

Our findings support association of higher SOS with lower arterial compliance (higher AD, lower ASI); that is, better bone health is associated with better vascular health. The relationship appeared consistent in men and women and by menopause status. There was no clear differential relationship by smoking status, diabetes, or BMI. A range of blood biomarkers were considered as potential mediators of the association between SOS and ASI; the mediation pattern appeared different for men and women and these markers did not adequately explain the observed associations. Higher SOS was associated with lower IHD mortality in men, but much less robustly in women. This relationship was not attenuated with addition of ASI to models, suggesting mediation through independent mechanisms. In summary, higher SOS was associated with better vascular health by ASI and AD and with lower IHD mortality in men; underlying mechanisms are complex and likely vary by sex.

### Strengths and limitations

The large, broad population sample in UKB permitted investigation of sex-specific, and in women menopause status-specific, relationships using validated measures of bone and cardiac health, incorporating a wide range of covariates and mediators. The age range in UKB was limited to 40 to 69 years at recruitment, as such, our results may not be applicable to younger or older ages. There is limited information in terms of the performance characteristics of the heel ultrasound device used in UK Biobank. Several instruments of the same type and same software version were used across the centers, but coefficients of variation and long-term stability data are not currently available. If anything, inability to accommodate these factors is likely to add noise and thus bias toward the null rather than generate any spurious relationships. Finally, we cannot exclude the possibility of residual confounding due to the observational design of the study, and it should be recognized that although mediation analysis partitions variance in the associations, we cannot conclude causal relationships directly from this analysis.

### Comparison with existing literature

Several smaller studies have investigated the relationship between bone quality and arterial stiffness made by pulse waveform analysis, but none using AD. In general, there is underrepresentation of men and premenopausal women and there are scant data through which to infer sex or menopausal status specific effects. A number of studies have been conducted in small highly selective cohorts with specific risk profiles, which limits the generalizability of their findings and has the inherent potential to introduce bias.

Consistent with our findings, in a study of 7865 Japanese men and women, Hirose and colleagues^(26)^ report a significant association between better bone quality on calcaneal QUS and lower arterial stiffness by pulse wave velocity (PWV), with the relationship appearing stronger for postmenopausal women. Avramovski and colleagues^(27)^ and Zhang and colleagues^(28)^ also report significant negative associations between BMD and arterial stiffness. Distinctions between men and women or menopause status were not considered, perhaps due to sample size limitations. These findings are consistent with results from small cohorts of Korean^(29)^ and Turkish women.^(30)^ In a study of 633 individuals, Giallauria and colleagues^(31)^ report a significant association between higher bone quality assessed by computed tomography and lower arterial stiffness by PWV for women, but not for men.

Several studies investigated the relationship between bone quality and vascular health in populations with specific risk profiles. For instance, Masugata and colleagues^(32)^ demonstrated a negative association between BMD and arterial stiffness in 52 hypertensive men and women. Interestingly, Li and colleagues^(33)^ documented a negative association between BMD and arterial stiffness by PWV in hypertensive men (*n* = 355), but not in a comparator group without hypertension. Similarly, Li and colleagues^(34)^ reported a significant negative association between lumbar spine BMD and arterial stiffness by PWV in 334 men with silent brain infarction, but not in 368 matched controls. Van Dijk and colleagues^(35)^ identified no association between arterial stiffness measures and BMD or QUS parameters in 519 older men and women with hyperhomocysteinemia. The findings to date from these generally small studies are therefore somewhat variable, but in general support the notion of positive associations between bone and cardiovascular health.

Our findings, in the largest sample studied to date, confirm the association of better bone quality (higher SOS) with lower arterial compliance (lower ASI, higher AD) with consistent relationships by sex and menopause status.

A number of studies have examined the relationship between serum markers of bone metabolism and arterial stiffness. In a study of the relationship between plasma regulators of bone metabolism in 1003 individuals with type 2 diabetes, Sharif and colleagues^(36)^ identified significant association between higher levels of plasma osteopontin and greater arterial stiffness. In a study of 144 postmenopausal women, Albu and colleagues^(37)^ identified significant association between higher plasma osteoprotegerin levels and greater arterial stiffness on PWV, but not with osteopontin (as per Sharif and colleagues^(36)^), suggesting possible differences in pathophysiology in men and women. However, given that osteoprotegerin and osteopontin have been implicated directly in vascular pathology as well as bone metabolism, such findings do not necessarily demonstrate direct bone-heart mechanisms.^(38)^ Indeed, such observations clearly demonstrate the complexity in these relationships and the difficulty in elucidating specific mechanisms in bone versus those in the vascular endothelium at the whole organ level. Our analysis of the effect of a range of blood biomarkers in mediating the relationship between SOS and ASI also suggested differences in mechanistic pathways in men and women.

Our finding of association of higher SOS with lower IHD mortality in men is consistent with previous reports of associations between lower BMD and greater riskof cardiovascular mortality outcomes, which, similar to our findings, appeared stronger in men.^(39–41)^ However several small studies in selected populations of older women,^(42,43)^ and a larger study,^(44)^ have demonstrated similar associations in women. In this latter investigation, Bauer and colleagues^(44)^ examined associations between baseline heel QUS (bone ultrasound attenuation, which is linearly highly correlated with speed of sound) and incident mortality. Among 5816 women, mean age 70 years, risk of cardiovascular death was 19% greater for each 1 SD lower baseline BUA. It is notable the women assessed in these studies tended to be older than our population, and given that in the present analysis the point estimate for the SOS-mortality relationship in women was <1, it may well be that we had limited capacity to discern a female specific effect given their younger age and fewer events compared with these other studies (and indeed compared with the men in the present analysis). Interestingly, in the present study, the observed inverse association between SOS and IHD mortality in men was not explained by association of higher SOS with lower arterial compliance, suggesting alternative mediating mechanisms are relevant here.

## Conclusions

Our findings support a positive association between bone and vascular health measures that is consistent in men and women and with menopause. The association of higher SOS with lower IHD mortality appeared more robust for men than women and was not explained by associations with arterial stiffness. The underlying pathophysiology of the bone heart axis is complex and multifaceted and likely varies in men and women. Further research into potential mechanistic pathways is needed.

## Supplementary Material

Supplementary Table 1-11

## Figures and Tables

**Table 1:** Baseline Participant Characteristics (Baseline Sample)

Characteristic	Whole cohort (n = 159,542)	Men (*n* = 71,949)	Women (*n* = 87,593)
Age (years), median [IQR]	58 [50–63]	59 [51 –64]	58 [50–63]
Ethnicity (white-Caucasian), *n* (%)	145,627 (91.9)	65,804 (92.2)	79,823 (91.6)
Townsend deprivation score, median [IQR]	–1.8 [–3.4 to 0.8]	–1.8 [–3.4 to 0.8]	–1.8 [–3.4 to 0.7]
BMI (kg/m^2^), median [IQR]	26.7 [24.1 –29.8]	27.2 [24.9–29.9]	26.1 [23.4–29.8]
Current smoking, *n* (%)	16,085 (10.1)	8637 (12.0)	7448 (8.5)
Regular alcohol use, *n* (%)	67,664 (42.5)	36,478 (50.9)	31,186 (35.7)
Physical activity (metabolic equivalent minutes/week), median [IQR]	1891 [874–3786]	1908 [864–3930]	1866 [878–3666]
Multimorbidity (number of non-cancer illnesses), median [IQR]	2.0 [1.0–3.0]	2.0 [1.0–3.0]	2.0 [1.0–3.0]
Hypertension, *n* (%)	44,626 (28.0)	23,676 (32.9)	20,950 (23.9)
Diabetes, *n* (%)	8981 (5.6)	5351 (7.4)	3630 (4.1)
Hypercholesterolemia, *n* (%)	31,465 (19.7)	18,571 (25.8)	12,894 (14.7)
Postmenopausal, *n* (%)	–	–	53,940 (73.0)
Arterial stiffness index (m/s), median [IQR]	9.0 [6.9–11.2]	9.8 [7.8–11.8]	8.3 [15.3–15.7]
Speed of sound (10^2^ m/s), mean ± SD	15.5 (0.3)	15.6 (0.3)	15.5 (0.3)

Data are based on information collected at baseline assessment. Continuous variables are presented as median [IQR] or mean ± SD. Discrete data are presented as *n* (%).

**Table 2:** Linear Regression Models Showing Association of SOS With Measures of Arterial Stiffness in Men and Women

Parameter	Model 1: Age	Model 2: Age, exercise, smoking, deprivation, alcohol	Model 3: Model 2 + hypercholesterolemia, diabetes, hypertension
ASI			
Men (*n* = 71,949)			
B (95% CI)	–0.030 (–0.037 to –0.023)	–0.021 (–0.028 to –0.013)	–0.020 (–0.028 to –0.012)
*p*	4.8 × 10^–17^*	1.5 × 10^–7^*	2.6 × 10^–7^*
Women (*n* = 87,593)			
B (95% CI)	–0.027 (–0.034 to –0.021)	–0.024 (–0.031 to –0.016)	–0.026 (–0.033 to –0.018)
*p*	5.6 × 10^–16^*	6.0 × 10^–10^*	2.8 × 10^–11^*
*p* for interaction	.605	.541	.307
AD (ascending aorta)			
Men (*n* = 8767)			
B (95% CI)	0.018 (0.000–0.036)	0.017 (–0.002 to 0.036)	0.017 (–0.002 to 0.036)
*p*	.046	.085	.085
Women (*n* = 9462)			
B (95% CI)	0.025 (0.008–0.042)	0.020 (0.000–0.039)	0.020 (0.000–0.039)
*p*	.004*	.045*	.043*
*p* for interaction	.588	.846	.829
AD (descending aorta)			
Men (*n* = 8767)			
B (95% CI)	0.040 (0.021 –0.059)	0.037 (0.018–0.057)	0.037 (0.017–0.056)
*p*	2.2 × 10^–5^*	.0002*	.0002*
Women (n = 9462)			
B (95% CI)	0.017 (–0.000 to 0.035)	0.019 (–0.001 to 0.039)	0.019 (–0.000 to 0.039)
*p*	.057	.063	.054
*p* for interaction	.081	.194	.217

B = increase (number of SDs) in outcome for a 1-SD increase in SOS. AD = aortic distensibility; ASI = arterial stiffness index; B = beta coefficient; CI = confidence interval; SOS = speed of sound.

* *p* < .05.

**Table 3:** Linear Regression Models Showing Association of SOS With Measures of Arterial Stiffness in Women Stratified by Menopause Status

Parameter	Model 1: Age	Model 2: Age, exercise, smoking, deprivation, alcohol.	Model 3: Model 2+ hypercholesterolemia, diabetes, hypertension
ASI			
Premenopause (*n* = 33,653)			
B (95% CI)	–0.026 (–0.041 to –0.011)	–0.025 (–0.041 to –0.008)	–0.025 (–0.042 to –0.009)
^*p*^	.0008*	.003*	.003*
Postmenopause (n = 53,940)			
B (95% CI)	–0.019 (–0.028 to –0.010)	–0.015 (–0.025 to –0.005)	–0.018 (–0.028 to –0.007)
*p*	4.9 ×10^–5^*	.005*	.0009*
*p* for interaction	.433	.327	.449
AD (ascending aorta)			
Premenopause (*n* = 4333)			
B (95% CI)	0.016 (–0.017 to 0.049)	0.012 (–0.024 to 0.048)	0.012 (–0.024 to 0.048)
*p*	.338	.522	.516
Postmenopause (*n* = 5129)			
B (95% CI)	0.013 (–0.011 to 0.037)	0.011 (–0.016 to 0.038)	0.012 (–0.015 to 0.039)
*p*	.288	.483	.370
*p* for interaction	.884	.964	.984
AD (descending aorta)			
Premenopause (*n* = 4333)			
B (95% CI)	0.003 (–0.029 to 0.036)	0.001 (–0.035 to 0.036)	0.001 (–0.035 to 0.036)
*p*	.837	.973	.957
Postmenopause (*n* = 5129)			
B (95% CI)	0.004 (–0.020 to 0.028)	0.008 (–0.019 to 0.035)	0.009 (–0.018 to 0.035)
*p*	.741	.563	.513
*p* for interaction	.977	.749	.727

B = increase (number of SDs) in outcome for a 1-SD increase in SOS. AD = aortic distensibility; ASI = arterial stiffness index; B = beta coefficient; CI = confidence interval; SOS = speed of sound.

* *p* < .05.

**Table 4:** Competing Risk Models of the Association of SOS with Incident AMI and IHD Mortality (*n* = 477,683)

Parameter	Model 1: Age	Model 2: Age, exercise, smoking, deprivation, alcohol	Model 3: Model 2 + hypercholesterolemia, diabetes, hypertension
Incident AMI			
Men (*n* = 214,410)			
SHR (95% CI)	0.96 (0.93–0.99)	0.99 (0.96–1.02)	0.99 (0.96–1.02)
*p*	.002*	.651	.658
Women (*n* = 263,273)			
SHR (95% CI)	0.97 (0.93–1.01)	1.03 (0.97–1.08)	1.00 (0.95–1.05)
*p*	.159	.352	.987
IHD mortality			
Men (*n* = 214,410)			
SHR (95% CI)	0.81 (0.77–0.85)	0.86 (0.81 –0.91)	0.86 (0.81 –0.91)
*p*	7.8 × 10^–15^*	9.9 × 10^–7^*	4.0 × 10^–7^*
Women (*n* = 263,273)			
SHR (95% CI)	0.92 (0.82–1.02)	0.91 (0.78–1.05)	0.86 (0.75–1.00)
*p*	.093	.184	.051

AMI = acute myocardial infarction; ASI = arterial stiffness index; CI = confidence interval; SHR = subdistribution hazard ratio; IHD = ischemic heart disease.

* *p* < .05.
