# Supplementary material for: Poor Bone Quality is Associated With Greater Arterial Stiffness: Insights From the UK Biobank
Source: J Bone Miner Res. Author manuscript; Available in PMC 2022 Aug 6. (PMC7613252; doi:10.1002/jbmr.4164)
Supplement: Supplementary Table 1-11 [file EMS151237-supplement-Supplementary_Table_1_11.docx]

**Supplementary Table 1. Baseline participant characteristics (Imaging sample)**

|  | Whole cohort  (*n*=18,229) | Men  (*n*=8,767) | Women  (*n*=9,462) |
| --- | --- | --- | --- |
| Age | 56 [49-61] | 57 [50-62] | 55 [48-60] |
| Ethnicity (Caucasian) | 17,701 (97.4%) | 8,485 (97.2%) | 9,216 (97.6%) |
| Townsend deprivation score | -2.7 [-3.9 to -0.7] | -2.7 [-4.0 to -0.7] | -2.6 [-3.9 to -0.7] |
| Body mass index kg/m^2^ | 26.0 [23.6 to 28.7] | 26.7 [24.5 to 29.0] | 25.2 [22.9 to 28.3] |
| Current smoking | 1,147 (6.3%) | 671 (7.7%) | 476 (5.0%) |
| Regular alcohol use | 5,069 (57.9%) | 4, 167 (44.0%) | 9,236 (50.7%) |
| Physical activity (metabolic equivalent minutes/week) | 1,733 [810 to 3,339] | 1,740 [809 to 3,394] | 1,714 [810 to 3,306] |
| Multimorbidity (number of non-cancer illnesses) | 1.0 [0.0 to 2.0] | 1.0 [0.0 to 2.0] | 1.0 [0.0 to 2.0] |
| Hypertension | 3,597 (18.7%) | 2,121 (24.2%) | 1,476 (15.6%) |
| Diabetes | 472 (2.6%) | 308 (3.5%) | 164 (1.7%) |
| Hypercholesterolaemia | 2,435 (13.4%) | 1.632 (18.6%) | 803 (8.5%) |
| Post-menopausal | – | – | 5,129 (64.4%) |
| Aortic distensibility at the ascending aorta (10^-3^ mmHg^-1^) | 1.2 [0.8-1.9] | 1.3 [0.8-1.9] | 1.2 [0.7-2.0] |
| Aortic distensibility at the descending aorta (10^-3^ mmHg^-1^) | 2.3 [1.8-2.9] | 2.2 [1.7-2.8] | 2.3 [1.8-2.9] |
| Speed of sound (10^2^m/s) | 15.6 (0.3) | 15.6 (0.3) | 15.5 (0.3) |

**Supplementary Table 1 footnote:** Data based on information collected at baseline assessment. Continuous variables presented as median [interquartile range] or mean (standard deviation). Discrete data presented as frequencies (percentages).

**Supplementary Table 2. Tests for non-linearity using cubic spline models with 5 knots showing p-values for non-linear vs linear models**

|  |  | Model 1: Age adjusted | Model 2: Age, exercise, smoking, deprivation, alcohol | Model 3: Model 2+ hypercholesterolaemia, diabetes, hypertension |
| --- | --- | --- | --- | --- |
|  | **ASI** | | | |
| Men | p-value | 0.354 | 0.117 | 0.099 |
| Women | p-value | 0.126 | 0.782 | 0.849 |
|  | **AD (ascending aorta)** | | | |
| Men | p-value | 0.909 | 0.755 | 0.823 |
| Women | p-value | 0.764 | 0.724 | 0.699 |
|  | **AD (descending aorta)** | | | |
| Men | p-value | 0.494 | 0.608 | 0.560 |
| Women | p-value | 0.393 | 0.640 | 0.654 |

**Supplementary Table 2 footnote:** AD: aortic distensibility; ASI: arterial stiffness index.

**Supplementary Table 3. Linear regression models showing association of SOS with measures of arterial stiffness stratified by sex and smoking status**

|  |  | Model 1: Age | Model 2: Age, exercise, deprivation, alcohol | Model 3: Model 2+ hypercholesterolaemia, diabetes, hypertension |  |
| --- | --- | --- | --- | --- | --- |
| Men | **ASI** | | | |  |
| Non-smokers | B (95% CI) | -0.023 (-0.030, -0.016) | -0.021 (-0.028, -0.013) | -0.020 (-0.028, -0.013) |  |
| n=63,312 | p-value | 8.8$\times$10^-11^* | 1.2$\times$10^-7^* | 2.1$\times$10^-7^* |  |
| Current smokers | B (95% CI) | -0.042 (-0.060, -0.024) | -0.037 (-0.058, -0.017) | -0.037 (-0.058, -0.016) |  |
| n=8,637 | p-value | 5.9$\times$10^-6^* | 0.0004* | 0.0004* |  |
| P value for interaction |  | 0.054 | 0.142 | 0.138 |  |
|  | **AD (ascending aorta)** | | | | |
| Non-smokers | B (95% CI) | 0.018 (0.001, 0.037) | 0.015 (-0.004, 0.034) | 0.015 (-0.004, 0.034) |  |
| n=8,096 | p-value | 0.040* | 0.127 | 0.116 |  |
| Current smokers | B (95% CI) | 0.029 (-0.032, 0.090) | 0.039 (-0.025, 0.104) | 0.036 (-0.028, 0.101) |  |
| n=671 | p-value | 0.354 | 0.233 | 0.271 |  |
| P value for interaction |  | 0.754 | 0.474 | 0.539 |  |
|  | **AD (descending aorta)** | | | | |
| Non-smokers | B (95% CI) | 0.043 (0.023, 0.062) | 0.037 (0.016, 0.057) | 0.036 (0.016, 0.057) |  |
| n=8,096 | p-value | 1.6$\times$10^-5^* | 0.0005* | 0.0006* |  |
| Current smokers | B (95% CI) | 0.034 (-0.033, 0.100) | 0.040 (-0.031, 0.111) | 0.038 (-0.033, 0.108) |  |
| n=671 | p-value | 0.324 | 0.268 | 0.297 |  |
| P value for interaction |  | 0.793 | 0.928 | 0.969 |  |
| Women | **ASI** | | | |  |
| Non-smokers | B (95% CI) | -0.018 (-0.026, -0.011) | -0.020 (-0.029, -0.012) | -0.023 (-0.031, -0.014) |  |
| n=80,145 | p-value | 5.5$\times$10^-7^* | 1.7$\times$10^-6^* | 1.1$\times$10^-7^* |  |
| Current smokers | B (95% CI) | -0.026 (-0.050, -0.003) | -0.023 (-0.050, 0.004) | -0.026 (-0.053, 0.001) |  |
| n=7,448 | p-value | 0.025* | 0.096 | 0.056 |  |
| P value for interaction |  | 0.536 | 0.873 | 0.818 |  |
|  | **AD (ascending aorta)** | | | | |
| Non-smokers | B (95% CI) | 0.011 (-0.007, 0.030) | 0.007 (-0.013, 0.028) | 0.008 (-0.012, 0.028) |  |
| n=8,986 | p-value | 0.223 | 0.496 | 0.444 |  |
| Current smokers | B (95% CI) | 0.023 (-0.06, 0.107) | 0.005 (-0.087, 0.097) | 0.012 (-0.080, 0.103) |  |
| n=476 | p-value | 0.586 | 0.909 | 0.800 |  |
| P value for interaction |  | 0.786 | 0.970 | 0.936 |  |
|  | **AD (descending aorta)** | | | | |
| Non-smokers | B (95% CI) | 0.007 (-0.011, 0.025) | 0.009 (-0.011, 0.029) | 0.009 (-0.011, 0.029) |  |
| n=8,986 | p-value | 0.464 | 0.396 | 0.380 |  |
| Current smokers | B (95% CI) | -0.020 (-0.101, 0.062) | -0.028 (-0.118, 0.062) | -0.021 (-0.111, 0.069) |  |
| n=476 | p-value | 0.635 | 0.536 | 0.644 |  |
| P value for interaction |  | 0.533 | 0.429 | 0.521 |  |

**Supplementary Table 3 footnote:** ASI: arterial stiffness index; AD: aortic distensibility; B: beta coefficient; CI: confidence interval; SOS: speed of sound. B= increase (number of SDs) in outcome for a 1 SD increase in SOS. *indicates p-value <0.05.

**Supplementary Table 4. Linear regression models showing association of SOS with measures of arterial stiffness stratified by sex and diabetes status**

|  |  | Model 1: Age adjusted | Model 2: Age, exercise, smoking, deprivation, alcohol | Model 3: Model 2+ hypercholesterolaemia, hypertension |
| --- | --- | --- | --- | --- |
| Men | **ASI** |  |  |  |
| Non-diabetic | B (95% CI) | -0.033 (-0.040, -0.026) | -0.025 (-0.033, -0.018) | -0.025 (-0.032, -0.017) |
| n=66,598 | p-value | 6.4$\times$10^-22^* | 3.5$\times$10^-11^* | 6.8$\times$10^-11^* |
| Diabetic | B (95% CI) | -0.002 (-0.025, 0.021) | 0.009 (-0.017, 0.036) | 0.011 (-0.016, 0.037) |
| n=5,351 | p-value | 0.873 | 0.489 | 0.436 |
| P value for interaction |  | 0.012 | 0.014 | 0.012 |
|  | **AD (ascending aorta)** | | | |
| Non-diabetic | B (95% CI) | 0.019 (0.002, 0.037) | 0.015 (-0.004, 0.033) | 0.015 (-0.003, 0.034) |
| n=8,459 | p-value | 0.03* | 0.120 | 0.109 |
| Diabetic | B (95% CI) | 0.053 (-0.045, 0.151) | 0.071 (-0.031, 0.172) | 0.068 (-0.033, 0.169) |
| n=308 | p-value | 0.289 | 0.171 | 0.187 |
| P value for interaction |  | 0.504 | 0.286 | 0.312 |
|  | **AD (descending aorta)** | | | |
| Non-diabetic | B (95% CI) | 0.042 (0.023, 0.061) | 0.037 (0.017, 0.058) | 0.037 (0.017, 0.057) |
| n=8,459 | p-value | 0.00001* | 0.0003* | 0.0003* |
| Diabetic | B (95% CI) | 0.026 (-0.08, 0.134) | 0.013 (-0.101, 0.127) | 0.004 (-0.110, 0.118) |
| n=308 | p-value | 0.638 | 0.818 | 0.942 |
| P value for interaction |  | 0.773 | 0.684 | 0.574 |
| Women | **ASI** | | | |
| Non-diabetic | B (95% CI) | -0.026 (-0.033, -0.019) | -0.022 (-0.030, -0.013) | -0.023 (-0.031, -0.015) |
| n=83,963 | p-value | 2.1$\times$10^-12^* | 2.6$\times$10^-7^* | 3.5$\times$10^-8^* |
| Diabetic | B (95% CI) | -0.025 (-0.058, 0.007) | -0.020 (-0.060, 0.019) | -0.022 (-0.061, 0.018) |
| n=3,630 | p-value | 0.129 | 0.321 | 0.289 |
| P value for interaction |  | 0.981 | 0.943 | 0.936 |
|  | **AD (ascending aorta)** | | | |
| Non-diabetic | B (95% CI) | 0.012 (-0.006, 0.030) | 0.007 (-0.013. 0.027) | 0.008 (-0.012, 0.028) |
| n=9298 | p-value | 0.185 | 0.487 | 0.439 |
| Diabetic | B (95% CI) | 0.026 (-0.116, 0.169) | 0.013 (-0.161, 0.188) | 0.025 (-0.149, 0.199) |
| n=164 | p-value | 0.719 | 0.881 | 0.780 |
| P value for interaction |  | 0.850 | 0.945 | 0.850 |
|  | **AD (descending aorta)** | | | |
| Non-diabetic | B (95% CI) | 0.042 (0.021, 0.061) | 0.037 (0.017, 0.058) | 0.037 (0.017, 0.057) |
| n=9,298 | p-value | 0.00001* | 0.0003* | 0.0003* |
| Diabetic | B (95% CI) | 0.061 (-0.081, 0.203) | 0.048 (-0.125, 0.221) | 0.057 (-0.115, 0.229) |
| n=164 | p-value | 0.398 | 0.585 | 0.519 |
| P value for interaction |  | 0.773 | 0.684 | 0.574 |

**Supplementary Table 4 footnote:** ASI: arterial stiffness index; AD: aortic distensibility; B: beta coefficient; CI: confidence interval; SOS: speed of sound. B= increase (number of SDs) in outcome for a 1 SD increase in SOS. *indicates p-value <0.05.

**Supplementary Table 5. Linear regression models showing association of SOS with measures of arterial stiffness stratified by sex and BMI**

|  |  | Model 1: Age adjusted | Model 2: Age, exercise, smoking, social deprivation, alcohol | Model 3: Model 2+ hypercholesterolaemia, diabetes, hypertension |
| --- | --- | --- | --- | --- |
| Men | **ASI** | | | |
| Normal | B (95% CI) | -0.054 (-0.066, -0.041) | -0.037 (-0.050, -0.023) | -0.036 (-0.050, -0.023) |
| N=18501 | p-value | 2.3$\times$10^-17^* | 1.0$\times$10^-7^* | 1.7$\times$10^-7^* |
| Overweight | B (95% CI) | -0.040 (-0.050, -0.031) | -0.036 (-0.046, -0.025) | -0.035 (-0.046, -0.025) |
| N=35779 | p-value | 1.1$\times$10^-17^* | 8.1$\times$10^-12^* | 9.1$\times$10^-12^* |
| Obese | B (95% CI) | -0.004 (-0.017, 0.009) | 0.001 (-0.014, 0.015) | 0.001 (-0.014, 0.015) |
| N=17617 | p-value | 0.545 | 0.946 | 0.944 |
| P value for interaction |  | 2.1$\times$10^-7^ | 0.0006 | 0.0008 |
|  | **AD (ascending aorta)** | | | |
| Normal | B (95% CI) | 0.030 (-0.001, 0.061) | 0.028 (-0.005, 0.061) | 0.028 (-0.005, 0.061) |
| N=2641 | p-value | 0.059 | 0.095 | 0.098 |
| Overweight | B (95% CI) | 0.021 (-0.003, 0.044) | 0.013 (-0.012, 0.038) | 0.014 (-0.011, 0.038) |
| N=4534 | p-value | 0.301 | 0.310 | 0.279 |
| Obese | B (95% CI) | 0.003 (-0.039, 0.044) | 0.007 (-0.037, 0.052) | 0.006 (-0.038, 0.051) |
| N=1588 | p-value | 0.901 | 0.742 | 0.788 |
| P value for interaction |  | 0.352 | 0.474 | 0.461 |
|  | **AD (descending aorta)** | | | |
| Normal | B (95% CI) | 0.068 (0.034, 0.101) | 0.067 (0.031, 0.103) | 0.067 (0.032, 0.103) |
| N=2641 | p-value | 8.6$\times$10^-5^* | 0.0002* | 0.0002* |
| Overweight | B (95% CI) | 0.028 (0.002, 0.054) | 0.022 (-0.006, 0.049) | 0.020 (-0.007, 0.048) |
| N=4534 | p-value | 0.036* | 0.125 | 0.147 |
| Obese | B (95% CI) | 0.049 (0.004, 0.094) | 0.044 (-0.004, 0.092) | 0.042 (-0.006, 0.089) |
| N=1588 | p-value | 0.031 | 0.070 | 0.085 |
| P value for interaction |  | 0.322 | 0.269 | 0.233 |
| Women | **ASI** | | | |
| Normal | B (95% CI) | -0.035 (-0.045, -0.024) | -0.030 (-0.042, -0.018) | -0.030 (-0.042. -0.018) |
| N=34706 | p-value | 2.5$\times$10^-10^* | 1.2$\times$10^-6^* | 7.1$\times$10^-7^* |
| Overweight | B (95% CI) | -0.032 (-0.044, -0.021) | -0.024 (-0.038, -0.011) | -0.025 (-0.039, -0.012) |
| N=32011 | p-value | 4.9x10^-8^* | 0.0003* | 0.0002* |
| Obese | B (95% CI) | -0.024 (-0.039, -0.010) | -0.026 (-0.043, -0.009) | -0.027 (-0.044, -0.010) |
| N=20800 | p-value | 0.001* | 0.003* | 0.002* |
| P value for interaction |  | 0.262 | 0.657 | 0.687 |
|  | **AD (ascending aorta)** | | | |
| Normal | B (95% CI) | 0.012 (-0.013, 0.037) | 0.007 (-0.021, 0.034) | 0.008 (-0.019, 0.035) |
| N=4575 | p-value | 0.351 | 0.634 | 0.570 |
| Overweight | B (95% CI) | 0.007 (-0.023, 0.037) | 0.003 (-0.031, 0.036) | 0.003 (-0.031, 0.036) |
| N=3380 | p-value | 0.641 | 0.883 | 0.873 |
| Obese | B (95% CI) | 0.020 (-0.028, 0.067) | 0.013 (-0.042, 0.068) | 0.014 (-0.040, 0.069) |
| N=1501 | p-value | 0.421 | 0.638 | 0.614 |
| P value for interaction |  | 0.827 | 0.876 | 0.894 |
|  | **AD (descending aorta)** | | | |
| Normal | B (95% CI) | 0.003 (-0.022, 0.027) | -0.000 (-0.027, 0.027) | 0.001 (-0.026, 0.028) |
| N=4575 | p-value | 0.816 | 0.976 | 0.964 |
| Overweight | B (95% CI) | 0.008 (-0.022, 0.037) | 0.011 (-0.022, 0.044) | 0.011 (-0.022, 0.043) |
| N=3380 | p-value | 0.616 | 0.499 | 0.526 |
| Obese | B (95% CI) | -0.009 (-0.056, 0.037) | 0.012 (-0.041, 0.065) | 0.012 (-0.040,0.065) |
| N=1501 | p-value | 0.680 | 0.658 | 0.644 |
| P value for interaction |  | 0.797 | 0.556 | 0.585 |

**Supplementary Table 5 footnote:** ASI: arterial stiffness index; AD: aortic distensibility; B: beta coefficient; BMI: body mass index; CI: confidence interval; SOS: speed of sound. B= increase (number of SDs) in outcome for a 1 SD increase in SOS. Overweight =BMI >25; Obese=BMI >30 – as per World Health Organisation guidance. *indicates p-value <0.05.

**Supplementary Table 6. Indirect effect (mediated effect) of speed of sound on arterial stiffness index through each mediator in men, tested individually**

| Mediator | Beta | 95% CI | p-value | Percentage mediated |
| --- | --- | --- | --- | --- |
| Alkaline phosphatase | -0.003 | (-0.004, -0.002) | 1.5$\times$10^-18^* | 11.5% |
| Calcium | 0.000 | (0.000, 0.0001) | 0.45 | 0.6% |
| Phosphate | -0.001 | (-0.002, -0.001) | 0.00002* | 4.1% |
| Vitamin D | -0.001 | (-0.002, -0.001) | 1.2$\times$10^-9^* | 4.8% |
| C Reactive Protein | -0.003 | (-0.003, -0.002) | 9.3$\times$10^-13^* | 10.1% |
| Creatinine | -0.001 | (-0.001, 0.000) | 0.02 | 2.1% |
| IGF1 | -0.001 | (-0.002, -0.001) | 0.00002* | 4.0% |
| SHBG | 0.005 | (0.004, 0.006) | 3.4$\times$10^-25^* | -17.2% |
| Testosterone | -0.002 | (-0.002, -0.001) | 1.1$\times$10^-11^* | 6.7% |
| Oestradiol | 0.000 | (0.000, 0.001) | 0.493 | -0.6% |
| Cystatin C | -0.001 | (-0.001, 0.000) | 0.00008* | 2.8% |
| Testosterone/SHBG | 0.003 | (0.002, 0.004) | 10.0$\times$10^-10^* | -11.9% |
| Hypertension | -0.0004 | (-0.001, -0.00008) | 0.011 | 1.6% |
| Hypercholesterolaemia | 0.000 | (0.000, 0.00004) | 0.839 | 0.0% |
| Diabetes | 0.000 | (0.000, 0.0002) | 0.088 | -0.4% |

**Supplementary Table 6 footnote:** CI: confidence interval; IGF1: insulin like growth factor 1; SHBG: Sex hormone binding globulin. Significance level p<0.003. *indicates p-value<0.003.

**Supplementary Table 7. Indirect effect (mediated effect) of speed of sound on arterial stiffness index through each mediator in women, tested individually**

| Mediator | Beta | 95% CI | p-value | Percentage mediated |
| --- | --- | --- | --- | --- |
| Alkaline phosphatase | -0.004 | (-0.005, -0.004) | 4.7$\times$10^-30^* | 23.8% |
| Calcium | -0.0002 | (-0.0004, -0.0001) | 0.012 | 0.0% |
| Phosphate | -0.003 | (-0.003, -0.002) | 9.9$\times$10^-15^* | 13.6% |
| Vitamin D | 0.000 | (0.000, 0.001) | 0.303 | 0.0% |
| C Reactive Protein | 0.003 | (0.002, 0.004) | 1.4$\times$10^-12^* | -14.3% |
| Creatinine | 0.000 | (0.000, 0.0002) | 0.57 | 0.0% |
| IGF1 | -0.0002 | (-0.0004, 0.000) | 0.028 | 0.0% |
| SHBG | 0.006 | (0.005, 0.007) | 4.4$\times$10^-32^* | -27.3% |
| Testosterone | -0.0001 | (0.000, 0.0004) | 0.182 | 0.0% |
| Oestradiol | -0.0001 | (0.000, 0.0002) | 0.576 | 0.0% |
| Cystatin C | -0.001 | (-0.001, -0.0002) | 0.007 | 4.8% |
| Testosterone/SHBG | 0.003 | (0.002, 0.004) | 1.9$\times$10^-10^* | -12.0% |
| Hypertension | 0.002 | (0.001, 0.002) | 5.1$\times$10^-14^* | -5.0% |
| Hypercholesterolaemia | 0.0004 | (0.000, 0.0007) | 0.001* | -2.0% |
| Diabetes | 0.0008 | (0.001, 0.0012) | 9.9$\times$10^-6^* | -4.0% |

**Supplementary Table 7 footnote:** CI: confidence interval; IGF1: insulin like growth factor 1; SHBG: Sex hormone binding globulin. Significance level p<0.003. *indicates p-value<0.003.

**Supplementary Table 8. Independent indirect effect (mediated effect) of speed of sound on arterial stiffness index through each mediator in men**

| Mediator | Beta | 95% CI | p-value | Percentage mediated |
| --- | --- | --- | --- | --- |
| Alkaline phosphatase | -0.0021 | (-0.0028, -0.0015) | 6.6$\times$10^-11^ | 7.5% |
| Phosphate | -0.0013 | (-0.0018, -0.0008) | 1.8$\times$10^-7^ | 4.6% |
| Vitamin D | -0.0009 | (-0.0012, -0.0005) | 0.00001 | 3.2% |
| C Reactive Protein | -0.0017 | (-0.0023, -0.0012) | 1.4$\times$10^-9^ | 6.1% |
| IGF1 | -0.0016 | (-0.0022, -0.001) | 7.0$\times$10^-8^ | 5.7% |
| SHBG | 0.0048 | (0.0032, 0.0064) | 3.2$\times$10^-9^ | -17.1% |
| Testosterone | 0.001 | (0.0005, 0.0014) | 0.00009 | -3.6% |
| Cystatin C | -0.0004 | (-0.0006, -0.0001) | 0.004 | 1.4% |

**Supplementary Table 8 footnote:** CI: confidence interval; IGF1: insulin like growth factor 1; SHBG: Sex hormone binding globulin. Significance level p<0.006.

**Supplementary Table 9. Independent indirect effect (mediated effect) of speed of sound on arterial stiffness index through each mediator in women**

| Mediator | Beta | 95% CI | p-value | Percentage mediated |
| --- | --- | --- | --- | --- |
| Alkaline phosphatase | -0.0021 | (-0.0027, -0.016) | 3.1$\times$10^-13^ | 9.6% |
| Phosphate | -0.0029 | (-0.0036, -0.0022) | 4.1$\times$10^-17^ | 13.2% |
| C Reactive Protein | 0.0019 | (0.0013, 0.0025) | 2.1$\times$10^-9^ | -8.6% |
| SHBG | 0.0043 | (0.0035, 0.0051) | 1.6$\times$10^-27^ | -19.6% |
| Hypertension | 0.0007 | (0.0004, 0.0011) | 0.00003 | -5.5% |

**Supplementary Table 9 footnote:** CI: confidence interval; IGF1: insulin like growth factor 1; SHBG: Sex hormone binding globulin. Significance

**Supplementary Table 10. Baseline participant characteristics (IHD sample)**

|  | Men | | Women | |
| --- | --- | --- | --- | --- |
|  | No IHD  (*n*=212,688) | IHD  (*n*=1,722) | No IHD  (*n*=262,885) | IHD  (*n*=388) |
| Age | 58 [50 to 64] | 63 [58 to 66] | 58 [50 to 63] | 64 [60 to 67] |
| Ethnicity (Caucasian) | 201,008 (95.0%) | 1,631 (95.7%) | 248,596 (94.9%) | 371 (96.6%) |
| Townsend deprivation score | -2.2 [-3.7 to 0.6] | -1.0 [-3.0 to 2.3] | -2.2 [-3.7 to 0.4] | -0.9 [-3.0 to 2.0] |
| Body mass index kg/m^2^ | 27.2 [24.9 to 29.9] | 28.5 [25.9 to 32.0] | 26.1 [23.5 to 29.7] | 28.4 [25.0 to 32.7] |
| Current smoking | 26,295 (12.4%) | 456 (26.7%) | 23,297 (8.9%) | 83 (21.6%) |
| Regular alcohol use | 109,932 (51.8%) | 796 (46.4%) | 96,430 (36.8%) | 87 (22.7%) |
| Physical activity (metabolic equivalent minutes/week) | 1,862 [824 to 3,822] | 1,573 [582 to 3,506] | 1,765 [820-3492] | 1242 [506.5 to 3232.5] |
| Multimorbidity (number of non-cancer illnesses) | 1 [0-3] | 3 [2-5] | 1 [0-3] | 3 [2-5] |
| Hypertension | 68,407 (32.2%) | 1,053 (61.1%) | 63,260 (24.1%) | 232 (59.8%) |
| Diabetes | 14,326 (6.7%) | 419 (24.3%) | 9,840 (3.7%) | 91 (23.5%) |
| Hypercholesterolaemia | 51,304 (24.1%) | 934 (54.2%) | 36,669 (13.9%) | 178 (45.9%) |
| Post-menopausal | - | - | 159766 (72.3%) | 301 (93.7%) |
| Arterial stiffness index (m/s) | 9.8 [7.7 to 11.8] | 10.6 [8.5 to12.6] | 8.3 [6.3 to 10.5] | 9.6 [7.5 to 12.5] |
| Speed of sound (10^2^m/s) | 15.57 (0.30) | 15.50 (0.32) | 15.48 (0.29) | 15.41 (0.32) |

**Supplementary Table 10 footnote:** Data based on information collected at baseline assessment. Continuous variables presented as median [interquartile range] or mean (standard deviation). Discrete data presented as frequencies (percentages). IHD: ischaemic heart disease.

**Supplementary Table 11. Competing risk models of the association of SOS with incident AMI and IHD mortality**

|  |  | Model 2: Age, exercise, smoking, deprivation, alcohol + ASI | Model 3: Model 2+ hypercholesterolaemia, diabetes, hypertension+ ASI |
| --- | --- | --- | --- |
|  | **IHD mortality** |  |  |
| Men | SHR (95% CI) | 0.85 (0.76-0.95) | 0.85 (0.76-0.95) |
|  | p-value | 5.6$\times$10^-3^* | 3.5$\times$10^-3^* |
| Women | SHR (95% CI) | 0.77 (0.54-1.09) | 0.73 (0.51-1.03) |
|  | p-value | 0.134 | 0.076 |

**Supplementary Table 11 footnote:** AMI: acute myocardial infarction; ASI: arterial stiffness index; CI: confidence interval; IHD: ischaemic heart disease. SHR: subdistribution hazard ratio; SOS: speed of sound. *indicates p<0.05
